# Anti-Proliferative and Anti-Migratory Activity of Licorice Extract and Glycyrrhetinic Acid on Papillary Thyroid Cancer Cell Cultures

**DOI:** 10.3390/ijms251910800

**Published:** 2024-10-08

**Authors:** Jacopo Manso, Simona Censi, Maria Chiara Pedron, Loris Bertazza, Alberto Mondin, Edoardo Ruggeri, Susi Barollo, Chiara Sabbadin, Isabella Merante Boschin, Decio Armanini, Caterina Mian

**Affiliations:** 1 Endocrinology Unit, Department of Medicine (DIMED), University of Padua, 35128 Padua, Italy; 2 Endocrinology and Metabolism Unit, University Hospital S. Maria della Misericordia of Udine, 33100 Udine, Italy; 3 Department of Surgical, Oncological, and Gastroenterological Sciences, University of Padova, 35128 Padova, Italy

**Keywords:** thyroid, licorice, mineralocorticoid, glycyrrhetinic acid, thyroid cancer, papillary thyroid cancer

## Abstract

Papillary thyroid cancer (PTC) is the 8th most common cancer among women overall. Licorice contains over 300 active compounds, many of them with anti-cancer properties. Glycyrrhetinic acid (GA) is a major component of licorice. The aim of this study was to investigate the potential anti-proliferative effects of licorice and GA on PTC cell cultures. Licorice extract (LE) was produced from the root and tested on BCPAP and K1 cell lines, as well as GA and aldosterone. We used the MTT test to investigate the anti-proliferative activity, the wound healing test for the migratory activity, and finally, we analyzed cell cycle distribution, apoptosis, and oxidative stress after LE, GA, or aldosterone incubation. Both LE and GA reduced cell viability at 48 h and cell migration at 24 h in both PTC cultures. Aldosterone reduced cell migration only in K1 cells. LE and GA induced cell cycle arrest in the G0/G1 phase in the BCPAP cell line, while LE and aldosterone induced it in the K1 culture. GA but not LE increased the apoptosis rate in both cell lines, whereas LE but not GA increased oxidative stress in both cultures. This study presents the first evidence of the in vitro anti-proliferative and anti-migratory activity of LE and GA on PTC.

## 1. Introduction

Differentiated thyroid cancer includes papillary and follicular histotypes and accounts for the vast majority (>90%) of all thyroid cancers [1,2]. In the United States, the incidence of thyroid cancer has tripled, from 4.9 per 100,000 in 1975 to 14.3 per 100,000 in 2009 [3]. Papillary thyroid cancer (PTC) is the major contributor to the steadily increasing incidence of thyroid cancer. PTC is generally an indolent tumor, with an overall 10-year survival rate in the range of 85–90% [4,5,6].

Even nowadays, plants are very important for the development of new drugs. Historically, the use of plants in cancer treatment started way back and has been a primary resource for the discovery of traditional drugs now used in cancer treatment [7]. The use of the therapeutic effects of plants’ active compounds as potential anti-cancer drugs is a growing trend. Currently, more than half of the anti-cancer drugs are derived from plants, microorganisms, and marine organisms [8].

Licorice is one of the oldest and most used plants in traditional Chinese medicine. Glycyrrhiza uralensis Fisch., Glycyrrhiza inflata Bat., and Glycyrrhiza glabra L. are prescribed as licorice [9]. Licorice contains more than 20 triterpenoids and 300 flavonoids [9].

In recent years, many studies have proven that the active compounds isolated from licorice have anti-cancer, anti-microbial, anti-viral, anti-inflammatory, and immunoregulatory properties in vitro [9]. Several antitumor compounds isolated from licorice have been studied with in vitro models. Glycyrrhizin has been found to attenuate tumor necrosis factor-alpha (TNF-α) levels and induce apoptosis via mitochondrial and caspase-dependent pathways in colon cancer and leukemia [10,11]. Licochalcone A, an estrogenic flavonoid, stops cell cycle progression at the G2/M transition and induces apoptosis in gastric cancer [12], induces apoptosis via the phospholipase Cγ1-, Ca^2+^ pathway, and the reactive oxygen species pathway in human hepatocarcinoma cell cultures [13], and induces apoptosis via the Fasl-mediated caspase-dependent receptor pathway in oral cancer cell lines [14]. Glabridin, an estrogen receptor (ER) agonist, inhibits migration, invasion, and angiogenesis of human breast adenocarcinoma MDA-MB-231 cells by inhibiting the focal adhesion kinase/Rho-associated kinase (FAK/Rho) signaling pathway [15]. However, its effect on breast cancer cell growth is biphasic: at low concentrations, glabridin shows a stimulatory effect on cell growth dependent on ER binding, while at high concentrations, it has an ER-independent anti-proliferative effect [16]. Glycyrrhetinic acid (GA) induces apoptosis and cell cycle arrest in the G2 phase and down-regulates the expression of nuclear factor kappa-light-chain enhancer of activated B cells (NF-κB), vascular endothelial growth factor, and matrix metalloproteinase-9 (MMP-9) in prostate and gastric cancer cells [17,18].

Licorice has many endocrine effects. Particularly, prolonged consumption of licorice has been linked to the development of hypertension, a condition known as pseudohyperaldosteronism. The main constituent of the root is glycyrrhizic acid, which in the stomach and intestines is hydrolyzed to GA, which is responsible for pseudohyperaldosteronism both by blocking the enzyme 11β-Hydroxysteroid dehydrogenase type 2 and by a direct agonist effect on the mineralocorticoid receptor (MR) [19].

Aldosterone (Aldo) is the main mineralocorticoid hormone responsible for the regulation of fluid and electrolyte balance and blood pressure [20]. Aldo is secreted by the glomerulosa zone of the adrenal glands and is controlled by the renin–angiotensin–aldosterone system. Aldo acts on target cells through binding to its nuclear receptor, the MR [21]. Aldo exerts its action through genomic (in hours and days) and non-genomic (in seconds and minutes) effects. Aldo’s main target cells are epithelial cells, particularly in the kidney [22], but nonepithelial cells such as brain neurons [23], cardiomyocytes [24], or mononuclear nuclear leukocytes [25] are also considered non-classical targets of Aldo. Recently, we demonstrated the gene and protein expression of MR in both normal and cancer thyroid cells, identifying the thyroid as a new non-classical target tissue of mineralocorticoids [26]. We showed both gene and protein expression and functional activation of the MR in two human PTC cell models, the BCPAP and K1 cell lines. MR gene expression was 10-fold higher in BCPAP than in K1. This difference can be explained by their intrinsic differences, as the BCPAP cell line derives from a poorly differentiated PTC, while the K1 cell line derives from metastasis of a well-differentiated PTC [27], which also results in a different molecular substrate with BCPAP carrying *BRAF* (V600E) and *TP53* (D259Y) mutations, while the K1 cell line carries *BRAF* (V600E) and *PI3KCA* (E542K) mutations [28].

Given that, among the 320 licorice active compounds, the main ones are ER and MR agonists, both ER and MR are present in PTC, and the in vitro anti-cancer properties of licorice in other cancer cultures, the aim of this study was to investigate the possible anti-proliferative role of licorice and GA on PTC cell cultures.

## 2. Results

### 2.1. Cell Viability

**Figure 1:**
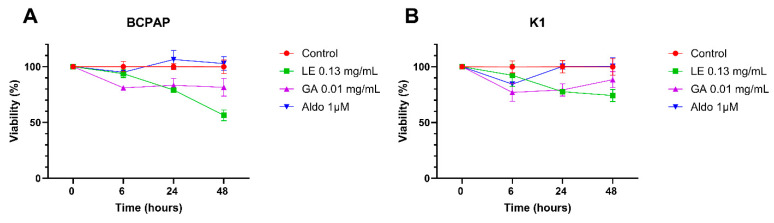
**MTT assay for BCPAP and K1 cells treated for 48 h**. (**A**) MTT assay in BCPAP cell line; (**B**) MTT assay in K1 cell line. Experiments were performed in triplicate and repeated three times. Aldo: Aldosterone; GA = Glycyrrhetinic acid; LE = Licorice extract; MTT assay: 3-(4,5-dimethylthiazol-2-yl)-2,5 diphenyltetrazolium bromide assay.

The effects of LE, GA, and Aldo on BCPAP and K1 cell viability were examined in vitro at 6, 24, and 48 h (Figure 1).

Compared to the control group, LE alone reduced cell viability to 56.4% in BCPAP (*p* < 0.001) and 74.2% in K1 (*p* = 0.0013) after 48 h. Similarly, after 48 h, the use of GA alone reduced cell viability to 81.5% in BCPAP (*p* = 0.01) and to 88.4% in K1 (not statistically significant). Aldosterone alone had no effect on the viability of the two cell lines.

### 2.2. Cell Migration

**Figure 2:**
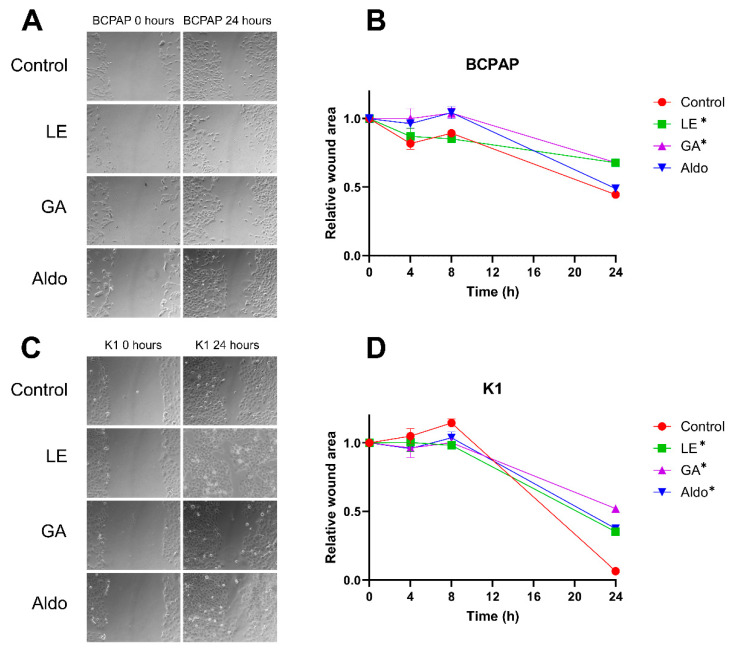
**Wound healing assay in papillary thyroid cancer cultures treated with licorice extract and glycyrrhetinic acid**. (**A**) Representative photos of the migration of the BCPAP cell line at time zero and after 24 h; (**B**) BCPAP relative wound area. Control vs. LE after 24 h * = *p* < 0.0001; Control vs. GA after 24 h * = *p* < 0.0001 (**C**) Representative photos of the migration of the K1 cell line at time zero and after 24 h; (**D**) K1 relative wound area. Control vs. LE after 24 h * = *p* < 0.0001; Control vs. GA after 24 h * = *p* < 0.0001; Control vs. Aldo after 24 h * = *p* < 0.0001. GA = Glycyrrhetinic acid 0.01 mg/mL; LE = Licorice extract 0.13 mg/mL; Aldo = Aldosterone 1 µM.

We examined the migratory potential of PTC cell lines after treatment with LE 0.13 mg/mL, GA 0.01 mg/mL, and Aldo 1 µM. Cell migration was assessed at multiple time points, specifically 4, 8, and 24 h after treatment. We observed a significant decrease in the area covered by cells in the BCPAP cell line when treated with LE and GA, compared with untreated cells (Figure 2, *p* < 0.0001).

Similarly, in the K1 cell line, tumor cell migration was significantly attenuated in the presence of LE, GA, and Aldo (*p* < 0.0001) compared with the control group.

### 2.3. Cell Cycle Analysis

**Figure 3:**
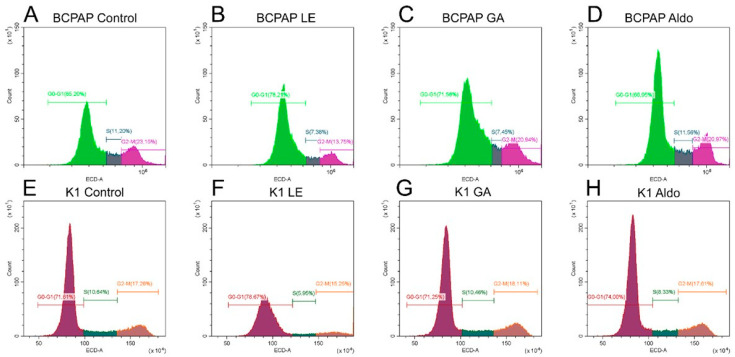
**Cell cycle distribution in papillary thyroid cancer cultures treated with licorice extract and glycyrrhetinic acid**. (**A**) BCPAP Control; (**B**) BCPAP treated with licorice extract 0.13 mg/mL; (**C**) BCPAP treated with glycyrrhetinic acid 0.01 mg/mL; (**D**) BCPAP treated with Aldosterone 1 µM; (**E**) K1 Control; (**F**) K1 treated with licorice extract 0.13 mg/mL; (**G**) K1 treated with glycyrrhetinic acid 0.01 mg/mL; (**H**) K1 treated with Aldosterone 1 µM.

We used flow cytometry to analyze the cell cycle distribution on the treated cells (Figure 3). In the BCPAP cell line, the percentage of cells in the G0/G1 phase was 65.20%. LE treatment increased this percentage to 78.21% and GA treatment to 71.58%. With Aldosterone alone, the percentage of cells in G0/G1 remained virtually unchanged (66.95%). In the K1 cell line, the percentage of cells in the G0/G1 phase was 71.61%. Again, LE treatment increased this percentage to 78.67%, while GA made no difference. With Aldosterone alone, the percentage of cells in G0/G1 increased to 74.00%.

### 2.4. Apoptosis Analysis

**Figure 4:**
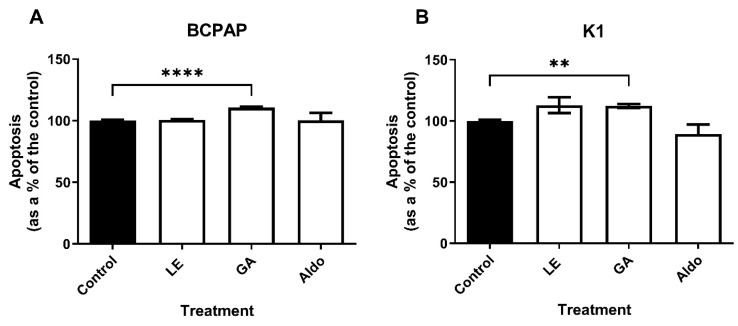
**Apoptosis analysis in papillary thyroid cancer cultures treated with licorice extract and glycyrrhetinic acid**. (**A**) Apoptosis analysis in BCPAP cell line; (**B**) Apoptosis analysis in K1 cell line. GA = glycyrrhetinic acid 0.01 mg/mL; LE = licorice extract 0.13 mg/mL; Aldo = Aldosterone 1 µM. Experiments were performed in triplicate. ** = *p* < 0.01; **** = *p* < 0.0001.

After 24 h of treatment, apoptosis detected by chemiluminescence assay revealed similar behavior in the two cell lines (Figure 4). In the BCPAP cell line, treatment with GA resulted in a significant 1.11-fold increase in apoptosis rate (*p* < 0.0001), while no significant effects were observed with LE. Similarly, in the K1 cell line, LE did not affect the level of caspases, while GA increased the apoptosis rate to 1.12-fold compared with the control group (*p* = 0.0012). Aldosterone had no effect on the caspase activity of both cell lines.

### 2.5. Oxidative Stress Analysis

**Figure 5:**
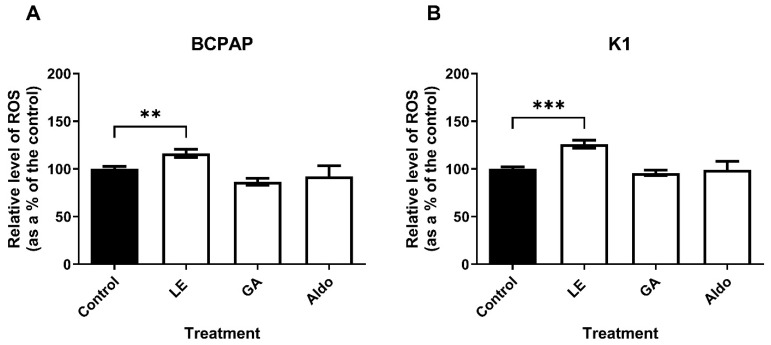
**Oxidative stress levels in papillary thyroid cancer cultures treated with compounds**. (**A**) Relative levels of ROS in BCPAP cell line; (**B**) Relative levels of ROS in K1 cell line. The cells were treated with the compounds for one hour. GA = glycyrrhetinic acid 0.01 mg/mL; LE = licorice extract 0.13 mg/mL; Aldo = Aldosterone 1 µM. Experiments were performed in triplicate. ** = *p* < 0.01; *** = *p* < 0.001.

As shown in Figure 5, LE treatment increased oxidative stress levels in both cell lines: in the BCPAP cell line by 1.16-fold (*p* = 0.0082) and in the K1 cell line by 1.26-fold (*p* = 0.0003), both compared with control. In contrast, GA and Aldo treatments in both cell lines did not affect reactive oxygen species (ROS) levels.

## 3. Discussion

Licorice has garnered significant scientific attention as a potential cancer treatment due to its ability to impact cancer cell cultures through various intracellular mechanisms, including apoptosis induction [12,14]. This is because of its numerous active compounds with anti-tumor activity. Given the high incidence of thyroid cancer in the population and its significant economic burden, the search for new drug treatments is an ongoing endeavor.

This study is the first to investigate the potential in vitro anti-tumor effect of licorice and its main component, GA, on thyroid cancer cells. A 2011 study by Chintharlapalli et al. demonstrated an anti-proliferative and pro-apoptotic effect of methyl 2-cyano-3,11-dioxo-18β-olean-1,12-dien-30-oate (CDODA-Me) and the corresponding 2-trifluoromethyl analog (CF3DODA-Me), two triterpenoids synthetically derived from GA, a major component of licorice [29]. They took among the various cell lines studied, one human anaplastic thyroid cancer and one K18 thyroid cancer cell line, and treated them with CDODA-Me and CF3DODA-Me, observing a reduction in both gene and protein expression of pituitary tumor-transforming gene-1 (PTTG-1). The downregulation of Sp proteins by CDODA-Me and CF3DODA-Me targeted PTTG-1, which is a known regulator of c-Myc and fibroblast growth factor-2 [29,30,31]. However, this study investigated the effect of two synthetic molecules derived from GA on thyroid cancer in vitro. Therefore, it did not properly examine the effect of licorice or its natural chemical constituents. Differently, we directly used LE and a single active compound naturally occurring in it, the GA. The LE contains up to 24% GA, primarily as a triterpenoid saponin glycoside of glycyrrhizic acid [32], similar to the high-performance liquid chromatography (HPLC) analysis of our LE.

In our experiments, overall, both LE and GA were able to inhibit cell survival and migratory potential in both PTC cultures, in agreement with the studies on other cancer cell lines. The observed cytotoxic effect of LE confirms the results of the study by Caroline and coworkers that showed an anti-proliferative effect of LE on oral cancer cells [33]. In addition, GA has been shown to inhibit cell survival in human gastric carcinoma cell cultures in a dose- and time-dependent manner [17]. It is not possible to identify a single molecular mechanism underlying the observed anti-proliferative and anti-migratory effects of LE and GA on PTC. This is probably the result of the complex interaction of the individual active components of licorice, mainly ER and MR agonists, with the different genomic (i.e., MR-driven) and non-genomic (i.e., non-MR-driven) pathways on BCAP and K1 cell lines.

Estrogen is a powerful growth factor for both benign and malignant thyroid cells, which could explain the higher prevalence of thyroid nodules and thyroid carcinoma in the female sex. However, unlike other cancers, the impact of estrogen on thyroid cancer is still uncertain [34]. The presence of ER has been demonstrated with different percentages in normal thyroid tissue, thyroid adenomas, multinodular goiter, and differentiated thyroid cancer [35]. Several studies have demonstrated the presence of ER in PTC, with percentages ranging from 40–67% [36]. In particular, ERα appears to exacerbate the development of PTC, while ERβ seems to play a protective role [37]. In the end, the prognostic impact of the presence of ER in PTC is unclear, with some studies in favor of more aggressive ER-positive PTC [38,39] and others against it [40]. In BCPAP and K1 cells, both ERα and ERβ have already been shown to be present, and estrogen has a stimulatory role, worsening the metastatic potential in vitro [41,42,43]. Thus, the opposite effect of LE on cell viability and migration that we observed in BCPAP and K1 cultures is likely attributable to the intricate interplay, both genomic and non-genomic, of the different multiple chemical constituents of LE rather than the singular active compounds such as GA, glabridin, and licochalcone A, which act as MR or ER agonists. Differently, since GA is a known MR agonist, we can speculate that its anti-proliferative and anti-migratory effect on PTC cultures could be related to its interaction with MR. However, the natural MR agonist, Aldo, exerted a migration-reducing effect exclusively in K1 cells. This suggests that the effect is probably not driven by the MR-driven genomic pathway alone, given that K1 cells possess 10-fold less MR gene expression than BCPAP, as demonstrated in our previous work [26]. Consequently, the impact of GA on PTC cells is a consequence of its multiple molecular interactions rather than solely as an agonist of MR.

Considering the anti-proliferative role of GA and the anti-migratory action of Aldo, the loss of mineralocorticoid signaling may provide an advantage to thyroid cancer cells. This is consistent with the observation of a progressive loss in MR expression in the more aggressive PTC histotypes [26].

Previous studies have reported that GA induces cell cycle arrest in the G1 phase in HepG2 cells, prostate cancer cells, and rat osteosarcoma cells [44,45,46]. This is concordant with what we observed in BCPAP cells treated with GA. Again, it can be reasonably inferred that the effect of GA on PTC cells is not due to its conventional action as an MR agonist, given that Aldo has been demonstrated to induce cell cycle arrest in G0/G1 in K1 cells (with less MR expression). In contrast, LE, which contains a multitude of active compounds, induces cell cycle arrest in the G0/G1 phase in both cell lines due to its multifaceted intracellular actions.

Regarding the induction of apoptosis, only GA showed a pro-apoptotic effect on both PTC cell lines, while LE did not. The potential apoptotic activity of GA was already demonstrated in gastric cancer culture [17]. Altogether, these findings suggest that GA may trigger PTC cell death through the apoptosis pathway. Conversely, only LE appeared to increase oxidative stress in both PTC cell lines, while GA and Aldo did not. Therefore, the anti-proliferative effect of LE appears to be at least partly mediated by an increase in ROS. It can be reasonably presumed that both the effects of GA on apoptosis and LE on oxidative stress are likely to be mediated by molecular mechanisms that are independent of their MR agonist action. This is based on the absence of effects being observed after incubation of PTC cultures with Aldo.

In conclusion, the present study demonstrated that LE and GA could both significantly inhibit PTC cell proliferation and migration via apoptosis or oxidative stress increase by molecular mechanisms not exclusively related to their genomic action as MR agonists.

The growing comprehension of the anti-tumor mechanisms of LE and GA suggests a future potential therapeutic application in thyroid cancer.

## 4. Materials and Methods

### 4.1. Cell Cultures and Maintenance

The BCPAP (human PTC cell line, RRID:CVCL_0153) was obtained from the German Collection of Microorganisms and Cell Cultures (Leibniz Institute DSMZ, Braunschweig, Germany); the K1 (human PTC cell line, RRID:CVCL_2537) was obtained from the European Collection of Authenticated Cell Cultures (ECACC, Sigma-Aldrich S.r.l., Milan, Italy). Testing for Mycoplasma infection was carried out on a monthly basis.

The BCPAP and K1 cell lines were cultured in RPMI 1640 (Gibco, Life Technologies, Carlsbad, CA, USA) supplemented with 10% fetal bovine serum (FBS) (Gibco), L-glutamine (2 mM), and penicillin/streptomycin (100 IU/mL and 100 μg/mL, respectively).

Adherent monolayer cultures were maintained in T75 culture flasks and incubated at 37 °C with 5% CO_2_ until they reached 85% confluency. Cells were detached using 0.025% trypsin (Sigma-Aldrich) and plated into T75 flasks at a density of 2 × 10^6^ cells.

### 4.2. Plant Material, Crude Extract and LC/MS Analysis

The extraction from the licorice root was performed, starting with a root weighing 7.53 g. The root underwent cryo-grinding to preserve the integrity of its phytoconstituents, achieved by rapid immersion in liquid nitrogen followed by grinding with a mortar to obtain a fine powder. The entire sample was collected in a 50 mL Falcon tube. The extraction process was carried out through maceration: 20 mL of 80% ethanol was added to the licorice powder, and the mixture was left under slow agitation for 24 h at 4 °C. The maceration product was then filtered in two steps: first, filtration was conducted using Whatman No. 1 filter paper with the assistance of a vacuum pump, followed by a second filtration with a 0.22 μm filter (Merck Millipore, Darmstadt, Germany) to sterilize the extract. The resulting filtrate was aliquoted into 1.5 mL Eppendorf tubes (500 µL per tube), and then the solvent was removed under reduced pressure using a temperature-controlled rotary vacuum evaporator (Rotavapor R-210, Flawil, Switzerland) until complete evaporation of the solvent.

The crude licorice extract (LE) was subsequently weighed and dissolved in dimethyl sulfoxide (DMSO, Sigma-Merck, Darmstadt, Germany) at a 1:10 weight/volume ratio for the complete product solubility. This LE was used for all experiments, divided into aliquots, and stored at −80 °C. After thawing, each aliquot was used only once.

The working concentration was determined through dose–response curves by 3-(4,5-dimethylthiazol-2-yl)-2,5 diphenyltetrazolium bromide (MTT) assay, using increasing doses of the LE at 24 h treatment time. The minimum dose that did not have an effect on viability was chosen (0.13 mg/mL), compared to the untreated control in both cell lines.

For the liquid chromatography–mass spectrometry (LC/MS) analysis, the following protocol was used: A total of 5 mg of powder was weighed and extracted with 2 mL of methanol. The solution was sonicated for 15 min and centrifuged for 10 min to deposit the non-solubilized parts. The supernatant was taken for chromatographic analysis. An Agilent Eclipse XDB C8 150 × 3 mm 3.5 µm column was used as the stationary phase, with a flow rate of 0.4 mL/min. The flow after the chromatographic column was split equally, with one part directed to the DAD and the other to the MS 500 mass spectrometer (Agilent/Varian), operating in negative mode and performing fragmentation. Several peaks showing UV spectra attributable to triterpenic saponins were detected in the LC-DAD chromatogram. Based on a comparison of fragmentation spectra, the literature, and reference standards, the species reported in Table 1 were identified and quantified. Supplementary Figure S1 shows the liquid chromatography–diode array detector (LC-DAD) chromatogram, the LC-MS chromatograms of the analyzed samples, the corresponding chromatographic traces, and some mass spectra related to *m*/*z* derivatives.

### 4.3. Drugs

18β-Glycyrrhetinic acid (GA, cat. no. G8503) and Aldosterone (Aldo, cat. no. A9477-5MG) were purchased from MERCK. The powders were dissolved in 10 mM stock solution in DMSO and stored at −80 °C. Given that many active compounds of licorice and thus GA are MR agonists, we decided to use Aldo to test whether the effect observed for LE and GA was somehow mediated by MR.

In our study, we chose the concentration of 0.01 mg/mL of GA and 1 µM of Aldo. As for the LE, the working concentration was determined through dose–response curves by MTT assay, using increasing doses of the compounds after 24 h of treatment. The minimum dose that did not have an effect on viability was chosen, compared to the untreated control in both cell lines.

### 4.4. Cell Viability

Cells were plated on 96-well tissue-culture microtiter plates at a density of 5 × 10^3^ cells per well. The next day, the cells were treated with LE, GA, and Aldo (LE 0.13 mg/mL, GA 0.01 mg/mL, Aldo 1 µM). We measured the effects on viability at different time points, from 6 to 48 h, using 3-(4,5-dimethylthiazol-2-yl)-2,5 diphenyltetrazolium bromide (MTT) assay (Sigma-Aldrich).

The MTT solution was directly added to the plate wells. After a 2-h incubation period, the culture media was removed, and 100 µL of DMSO was added to dissolve the formazan crystals. Absorbance was subsequently measured at 595 nm using a Viktor 3 Perkin-Elmer spectrophotometer. The signal intensity was then expressed as a percentage relative to the control. Experiments were conducted in triplicate and repeated three times.

### 4.5. Wound Healing

Cells were plated in six-well plates at a density of 1.2 × 10^5^ cells per well. The following day, the medium was removed, and a scratch wound was introduced using a pipette tip. The cells were then exposed to the drugs (LE at 0.13 mg/mL, GA at 0.01 mg/mL, and Aldo at 1 µM) for 24 h. Cell migration was observed over the subsequent 24 h using a Leica DMI6000CS microscope (Leica Microsystems, Wetzlar, Germany). The movement of cells into the wound area was recorded, and the average migration distance was measured using Leica Application Suite (LAS-AF) 3.1.1. software (Leica Microsystems, Buccinasco, Milan, Italy) and analyzed with ImageJ software (version 1.53e; Java 8 [64-bit]). All experiments were conducted in triplicate.

### 4.6. Cell Cycle Analysis

Cells were cultured in 12-well plates at a concentration of 1 × 10^6^ cells per well and exposed to the drugs for 24 h. Following treatment, cells were harvested using trypsin, rinsed with PBS, and fixed in 100% ethanol at 4 °C. After another wash with cold PBS (4 °C), the cells were treated with RNAse (1 mg/mL final concentration), resuspended in a propidium iodide (PI) solution (0.1 µg/mL in PBS), and incubated in the dark at 37 °C for 1 h. Subsequently, they were centrifuged, washed with PBS, and analyzed using a flow cytometer. The cell cycle was assessed with CytoFLEX (Beckman Coulter, Milan, Italy), and data files were exported and processed with CytExpert Software (Version 2.3, Beckman Coulter).

### 4.7. Apoptosis Analysis

Apoptosis was evaluated using the Caspase-Glo^®^ 3/7 assay, a luminescent and homogeneous method designed to detect caspase-3 and -7 activity, which are central enzymes in the apoptosis pathway. When the Caspase-Glo reagent is added, it induces cell lysis, allowing the caspases to cleave the provided substrate, leading to a luminescent signal produced by luciferase. The intensity of the luminescence correlates with the amount of active caspase present. Cells were cultured in a 96-well white plate and treated with the compounds as previously described. On the day of the experiment, the Caspase-Glo^®^ 3/7 reagent was prepared as per the manufacturer’s instructions and applied after 24 h of treatment. The plate was shaken at 500 rpm for 30 s, then incubated in darkness at room temperature for 90 min to stabilize the signal before luminescence was measured using a Viktor 3 Perkin-Elmer luminometer. The experiments were conducted in triplicate and repeated three times for consistency.

### 4.8. Reactive Oxygen Species-Glo H_2_O_2_ Assay

The evaluation of oxidative stress induced by our treatments was made through the Reactive Oxygen Species (ROS)-Glo™ H_2_O_2_ Assay (Promega, Madison, WI, USA; Cat. No. G8820) following the manufacturer’s instructions. Briefly, cells were seeded and treated for 1 h, as described before. Then, the H_2_O_2_ substrate was added to a final concentration of 25 µM. The plate was incubated for 90 min, and then 100 µL of ROS Glo detection solution was added. After 20 min at room temperature, luminescence was recorded using a luminometer (Viktor 3 Perkin-Elmer). Experiments were performed in triplicate and repeated three times.

### 4.9. Statistical Analysis

All statistical analyses were performed using the MedCalc^®^ Statistical Software (MedCalc Software Ltd., 2022, version 22.017, Ostend, Belgium) and GraphPad Prism (version 9.5.0). The Kolmogorov–Smirnov test was used to assess the normal distribution of all variables. Variables were compared by using Student’s *t*-test or two-way ANOVA. All results were considered statistically significant at *p* < 0.05.

## 5. Conclusions

This study presents the first evidence in the literature of the in vitro anti-proliferative and anti-migratory activity of LE and GA on PTC cultures. In the future, a better understanding of the molecular pathways associated with LE and GA action on cancer cells will provide new molecular targets, especially in thyroid cancer treatment.

## Figures and Tables

**Table 1:** High-performance liquid chromatography analysis of the main active compounds of licorice extract.

Retention Time (min)	Identification	[M-H]-	Fragmentation	Licorice Extract (mg/g)
14.3	Licorice saponine G2 isomer 1	837	775 661 351	0.80 ± 0.01
15	Licorice saponine G2 isomer 2	837	745 631 351	0.22 ± 0.01
15.6	Glycyrrhizin	821	759 645 351	5.51 ± 0.02
15.9	Licorice saponine B2	807	745 631 351	2.65 ± 0.02
16.4	Glycyrrhizinic acid isomer 1	821	759 645 351	0.09 ± 0.01
16.7	Licorice saponine B2 isomer	807	745 631 351	1.91 ± 0.01

## Data Availability

Data are available on request due to privacy restrictions.

## Supplementary Materials

The following supporting information can be downloaded at: https://www.mdpi.com/article/10.3390/ijms251910800/s1.

## References

[1] Siegel R., Ma J., Zou Z., Jemal A. (2014). Cancer statistics, 2014. CA Cancer J. Clin..

[2] Galuppini F., Pennelli G., Vianello F., Censi S., Zambonin L., Watutantrige-Fernando S., Manso J., Nacamulli D., Lora O., Pelizzo M.R. (2016). BRAF analysis before surgery for papillary thyroid carcinoma: Correlation with clinicopathological features and prognosis in a single-institution prospective experience. Clin. Chem. Lab. Med..

[3] Davies L., Welch H.G. (2014). Current thyroid cancer trends in the United States. JAMA Otolaryngol. Head Neck Surg..

[4] Haugen B.R., Alexander E.K., Bible K.C., Doherty G.M., Mandel S.J., Nikiforov Y.E., Pacini F., Randolph G.W., Sawka A.M., Schlumberger M. (2016). 2015 American Thyroid Association Management Guidelines for Adult Patients with Thyroid Nodules and Differentiated Thyroid Cancer: The American Thyroid Association Guidelines Task Force on Thyroid Nodules and Differentiated Thyroid Cancer. Thyroid.

[5] Eustatia-Rutten C.F., Corssmit E.P., Biermasz N.R., Pereira A.M., Romijn J.A., Smit J.W. (2006). Survival and death causes in differentiated thyroid carcinoma. J. Clin. Endocrinol. Metab..

[6] Barollo S., Pezzani R., Cristiani A., Redaelli M., Zambonin L., Rubin B., Bertazza L., Zane M., Mucignat-Caretta C., Bulfone A. (2014). Prevalence, tumorigenic role, and biochemical implications of rare BRAF alterations. Thyroid.

[7] Bahmani M., Shirzad H., Shahinfard N., Sheivandi L., Rafieian-Kopaei M. (2017). Cancer Phytotherapy: Recent Views on the Role of Antioxidant and Angiogenesis Activities. J. Evid. Based Complementary Altern. Med..

[8] Cragg G.M., Newman D.J. (2005). Plants as a source of anti-cancer agents. J. Ethnopharmacol..

[9] Yang R., Wang L.Q., Yuan B.C., Liu Y. (2015). The Pharmacological Activities of Licorice. Planta Med..

[10] Khan R., Khan A.Q., Lateef A., Rehman M.U., Tahir M., Ali F., Hamiza O.O., Sultana S. (2013). Glycyrrhizic acid suppresses the development of precancerous lesions via regulating the hyperproliferation, inflammation, angiogenesis and apoptosis in the colon of Wistar rats. PLoS ONE.

[11] Chueh F.S., Hsiao Y.T., Chang S.J., Wu P.P., Yang J.S., Lin J.J., Chung J.G., Lai T.Y. (2012). Glycyrrhizic acid induces apoptosis in WEHI-3 mouse leukemia cells through the caspase- and mitochondria-dependent pathways. Oncol. Rep..

[12] Xiao X.Y., Hao M., Yang X.Y., Ba Q., Li M., Ni S.J., Wang L.S., Du X. (2011). Licochalcone A inhibits growth of gastric cancer cells by arresting cell cycle progression and inducing apoptosis. Cancer Lett..

[13] Choi A.Y., Choi J.H., Hwang K.Y., Jeong Y.J., Choe W., Yoon K.S., Ha J., Kim S.S., Youn J.H., Yeo E.J. (2019). Correction to: Licochalcone A induces apoptosis through endoplasmic reticulum stress via a phospholipase Cγ1-, Ca^2+^-, and reactive oxygen species-dependent pathway in HepG2 human hepatocellular carcinoma cells. Apoptosis.

[14] Kim J.S., Park M.R., Lee S.Y., Kim D.K., Moon S.M., Kim C.S., Cho S.S., Yoon G., Im H.J., You J.S. (2014). Licochalcone A induces apoptosis in KB human oral cancer cells via a caspase-dependent FasL signaling pathway. Oncol. Rep..

[15] Hsu Y.L., Wu L.Y., Hou M.F., Tsai E.M., Lee J.N., Liang H.L., Jong Y.J., Hung C.H., Kuo P.L. (2011). Glabridin, an isoflavan from licorice root, inhibits migration, invasion and angiogenesis of MDA-MB-231 human breast adenocarcinoma cells by inhibiting focal adhesion kinase/Rho signaling pathway. Mol. Nutr. Food Res..

[16] Tamir S., Eizenberg M., Somjen D., Stern N., Shelach R., Kaye A., Vaya J. (2000). Estrogenic and antiproliferative properties of glabridin from licorice in human breast cancer cells. Cancer Res..

[17] Lin D., Zhong W., Li J., Zhang B., Song G., Hu T. (2014). Involvement of BID translocation in glycyrrhetinic acid and 11-deoxy glycyrrhetinic acid-induced attenuation of gastric cancer growth. Nutr. Cancer.

[18] Shetty A.V., Thirugnanam S., Dakshinamoorthy G., Samykutty A., Zheng G., Chen A., Bosland M.C., Kajdacsy-Balla A., Gnanasekar M. (2011). 18alpha-glycyrrhetinic acid targets prostate cancer cells by down-regulating inflammation-related genes. Int. J. Oncol..

[19] Sabbadin C., Bordin L., Dona G., Manso J., Avruscio G., Armanini D. (2019). Licorice: From Pseudohyperaldosteronism to Therapeutic Uses. Front. Endocrinol..

[20] Sabbadin C., Andrisani A., Ambrosini G., Bordin L., Dona G., Manso J., Ceccato F., Scaroni C., Armanini D. (2019). Aldosterone in Gynecology and Its Involvement on the Risk of Hypertension in Pregnancy. Front. Endocrinol..

[21] Balsamo A., Cicognani A., Gennari M., Sippell W.G., Menabo S., Baronio F., Riepe F.G. (2007). Functional characterization of naturally occurring NR3C2 gene mutations in Italian patients suffering from pseudohypoaldosteronism type 1. Eur. J. Endocrinol..

[22] Booth R.E., Johnson J.P., Stockand J.D. (2002). Aldosterone. Adv. Physiol. Educ..

[23] Patel P.D., Sherman T.G., Goldman D.J., Watson S.J. (1989). Molecular cloning of a mineralocorticoid (type I) receptor complementary DNA from rat hippocampus. Mol. Endocrinol..

[24] Pearce P., Funder J.W. (1987). High affinity aldosterone binding sites (type I receptors) in rat heart. Clin. Exp. Pharmacol. Physiol..

[25] Armanini D., Endres S., Kuhnle U., Weber P.C. (1988). Parallel determination of mineralocorticoid and glucocorticoid receptors in T- and B-lymphocytes of human spleen. Acta Endocrinol..

[26] Manso J., Pedron M.C., Mondin A., Censi S., Pennelli G., Galuppini F., Barollo S., Bertazza L., Radu C.M., Ghini F. (2024). First Evidence of Mineralocorticoid Receptor Gene and Protein Expression in Rat and Human Thyroid Tissues and Cell Cultures. Int. J. Mol. Sci..

[27] Affinito O., Orlandella F.M., Luciano N., Salvatore M., Salvatore G., Franzese M. (2022). Evolution of intra-tumoral heterogeneity across different pathological stages in papillary thyroid carcinoma. Cancer Cell Int..

[28] Saiselet M., Floor S., Tarabichi M., Dom G., Hebrant A., van Staveren W.C., Maenhaut C. (2012). Thyroid cancer cell lines: An overview. Front. Endocrinol..

[29] Chintharlapalli S., Papineni S., Lee S.O., Lei P., Jin U.H., Sherman S.I., Santarpia L., Safe S. (2011). Inhibition of pituitary tumor-transforming gene-1 in thyroid cancer cells by drugs that decrease specificity proteins. Mol. Carcinog..

[30] Pei L. (2001). Identification of c-myc as a down-stream target for pituitary tumor-transforming gene. J. Biol. Chem..

[31] Boelaert K., Yu R., Tannahill L.A., Stratford A.L., Khanim F.L., Eggo M.C., Moore J.S., Young L.S., Gittoes N.J., Franklyn J.A. (2004). PTTG’s C-terminal PXXP motifs modulate critical cellular processes in vitro. J. Mol. Endocrinol..

[32] Baltina L.A. (2003). Chemical modification of glycyrrhizic acid as a route to new bioactive compounds for medicine. Curr. Med. Chem..

[33] Caroline M.L., Muthukumar R.S., Priya A.H.H., Nachiammai N. (2023). Anticancer Effect of Plectranthus Amboinicus and Glycyrrhiza Glabra on Oral Cancer Cell Line: An Invitro Experimental Study. Asian Pac. J. Cancer Prev..

[34] Derwahl M., Nicula D. (2014). Estrogen and its role in thyroid cancer. Endocr. Relat. Cancer.

[35] Chaudhuri P.K., Prinz R. (1989). Estrogen receptor in normal and neoplastic human thyroid tissue. Am. J. Otolaryngol..

[36] Jalali-Nadoushan M.R., Amirtouri R., Davati A., Askari S., Siadati S. (2016). Expression of estrogen and progesterone receptors in papillary thyroid carcinoma. Caspian J. Intern. Med..

[37] Lu Y., Li J., Li J. (2016). Estrogen and thyroid diseases: An update. Minerva Med..

[38] Vannucchi G., De Leo S., Perrino M., Rossi S., Tosi D., Cirello V., Colombo C., Bulfamante G., Vicentini L., Fugazzola L. (2015). Impact of estrogen and progesterone receptor expression on the clinical and molecular features of papillary thyroid cancer. Eur. J. Endocrinol..

[39] Rubio G.A., Catanuto P., Glassberg M.K., Lew J.I., Elliot S.J. (2018). Estrogen receptor subtype expression and regulation is altered in papillary thyroid cancer after menopause. Surgery.

[40] Sturniolo G., Zafon C., Moleti M., Castellví J., Vermiglio F., Mesa J. (2016). Immunohistochemical Expression of Estrogen Receptor-α and Progesterone Receptor in Patients with Papillary Thyroid Cancer. Eur. Thyroid J..

[41] Dong W., Zhang H., Li J., Guan H., He L., Wang Z., Shan Z., Teng W. (2013). Estrogen Induces Metastatic Potential of Papillary Thyroid Cancer Cells through Estrogen Receptor α and β. Int. J. Endocrinol..

[42] Liu J., Xu T., Ma L., Chang W. (2021). Signal Pathway of Estrogen and Estrogen Receptor in the Development of Thyroid Cancer. Front. Oncol..

[43] Yang S., Gong Z., Liu Z., Wei M., Xue L., Vlantis A.C., Zhang Y., Chan J.Y., van Hasselt C.A., Zeng X. (2021). Differential Effects of Estrogen Receptor Alpha and Beta on Endogenous Ligands of Peroxisome Proliferator-Activated Receptor Gamma in Papillary Thyroid Cancer. Front. Endocrinol..

[44] Satomi Y., Nishino H., Shibata S. (2005). Glycyrrhetinic acid and related compounds induce G1 arrest and apoptosis in human hepatocellular carcinoma HepG2. Anticancer Res..

[45] Hawthorne S., Gallagher S. (2008). Effects of glycyrrhetinic acid and liquorice extract on cell proliferation and prostate-specific antigen secretion in LNCaP prostate cancer cells. J. Pharm. Pharmacol..

[46] Rabbitt E.H., Lavery G.G., Walker E.A., Cooper M.S., Stewart P.M., Hewison M. (2002). Prereceptor regulation of glucocorticoid action by 11beta-hydroxysteroid dehydrogenase: A novel determinant of cell proliferation. FASEB J..

