# Supplementary material for: Anti-Proliferative and Anti-Migratory Activity of Licorice Extract and Glycyrrhetinic Acid on Papillary Thyroid Cancer Cell Cultures
Source: Int J Mol Sci. 2024 Oct 8;25(19):10800. doi: 10.3390/ijms251910800 (PMC11476458; doi:10.3390/ijms251910800)
Supplement: Supplementary file 1 [file ijms-25-10800-s001.zip › ijms-3227618-supplementary.pdf]

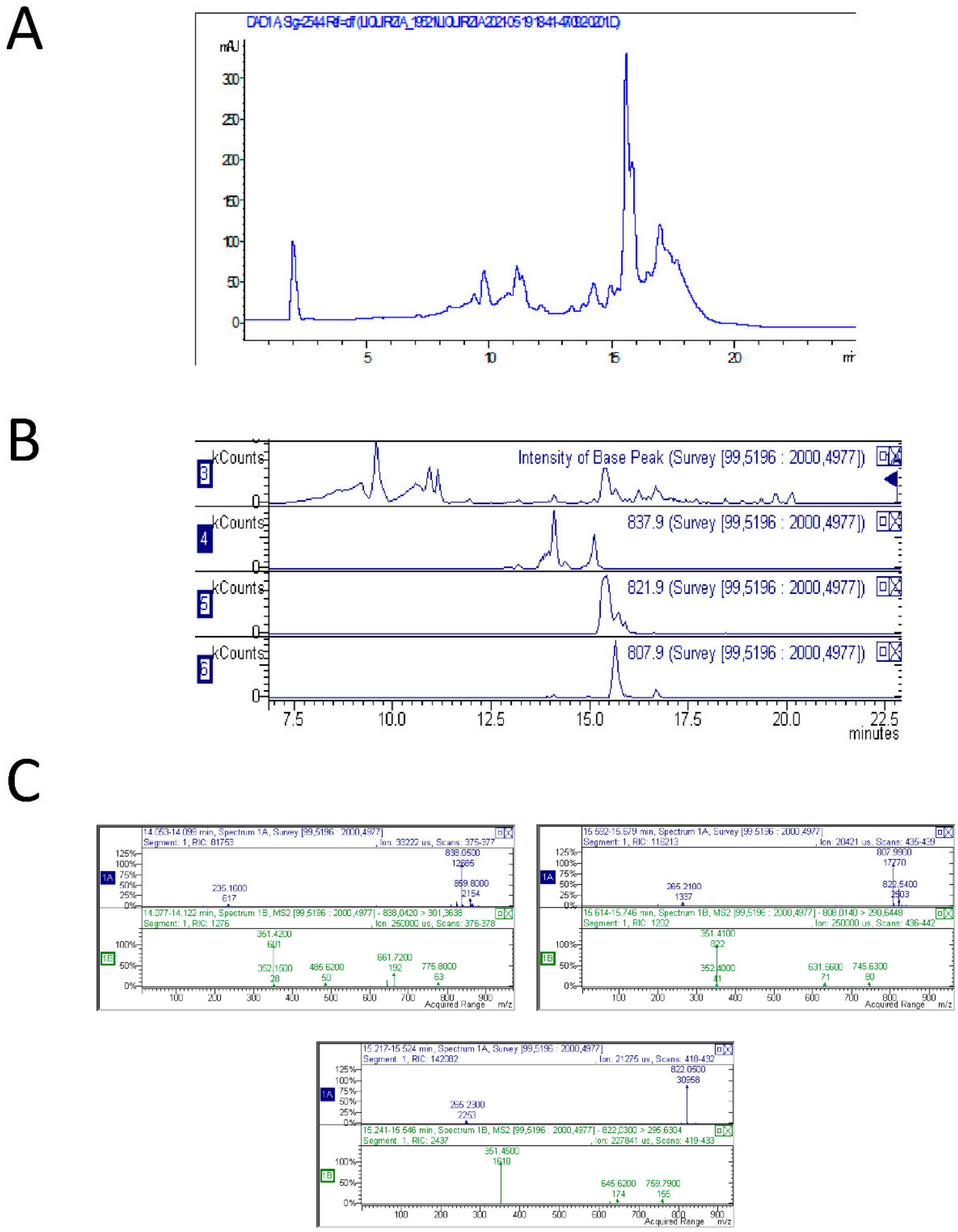

Supplemental Figure S1: LC/MS analysis of the licorice extract. (A) Exemplary LC-DAD chromatogram of the LE sample at 254 nm; (B) LC-MS chromatograms of the analyzed samples and corresponding chromatographic traces attributable to the derivatives listed in Table 1; (C) examples of some mass spectra related to the m/z derivatives.
